# Semantic Aware Stitching for Panorama

**DOI:** 10.3390/s24113512

**Published:** 2024-05-29

**Authors:** Yuan Jia, Zhongyao Li, Lei Zhang, Bin Song, Rui Song

**Affiliations:** 1 School of Telecommunications Engineering, Xidian University, Xi’an 710126, China; jiayuan@xidian.edu.cn (Y.J.); 21011220594@stu.xidian.edu.cn (Z.L.); 13820772721@163.com (L.Z.); 2 State Key Laboratory of Integrated Service Networks, Xidian University, Xi’an 710126, China

**Keywords:** superpixel, multi-image stitching, graph cut, artifact removal, semantics

## Abstract

The most critical aspect of panorama generation is maintaining local semantic consistency. Objects may be projected from different depths in the captured image. When warping the image to a unified canvas, pixels at the semantic boundaries of the different views are significantly misaligned. We propose two lightweight strategies to address this challenge efficiently. First, the original image is segmented as superpixels rather than regular grids to preserve the structure of each cell. We propose effective cost functions to generate the warp matrix for each superpixel. The warp matrix varies progressively for smooth projection, which contributes to a more faithful reconstruction of object structures. Second, to deal with artifacts introduced by stitching, we use a seam line method tailored to superpixels. The algorithm takes into account the feature similarity of neighborhood superpixels, including color difference, structure and entropy. We also consider the semantic information to avoid semantic misalignment. The optimal solution constrained by the cost functions is obtained under a graph model. The resulting stitched images exhibit improved naturalness. Extensive testing on common panorama stitching datasets is performed on the algorithm. Experimental results show that the proposed algorithm effectively mitigates artifacts, preserves the completeness of semantics and produces panoramic images with a subjective quality that is superior to that of alternative methods.

## 1. Introduction

Panoramic compositing is a key technique in virtual reality, autonomous driving and smart city applications. As a pioneering work, the AutoStitch algorithm proposed by Brown [1] provided a comprehensive computational process for generating stitched images aligning via the SIFT algorithm [2]. After years of studies, there are still some unsolved problems. The stitched images often exhibit misalignment and artifacts due to the lack of a homography condition between images. Additionally, issues such as lens distortion of the cameras, object motion and significant depth gaps among different objects can contribute to this misalignment. To address the limitations of global mapping, local transform matrices were added [3,4,5,6,7,8,9]. Some researchers divided the images into grids and fine-tuned the global mapping matrix using grid image patches as local units. To address distortion issues, some algorithms introduced similarity or geometry constraints [10,11,12,13] to ensure consistency in the texture of the images, but still based on the grid patches. Since the grid partitioning process only depends on the image resolution and the set grid size, it completely ignores the semantic information of objects in the images, resulting in noticeable semantic errors in some areas after stitching.

With the popularity of deep learning, it has been applied in some stitching tasks [14,15,16,17,18,19,20,21,22]. Learning-based stitching methods have realized automatic feature learning, end-to-end training and global information synthesis through deep learning networks, improving the robustness and generalization ability of image stitching, especially when dealing with complex scenarios. However, learning-based methods tend to cause blurring in the presence of large artifacts, and many algorithms can only handle the stitching of two images. When more than three images are involved, severe deformation will occur.

Another method to eliminate the artifacts is by using a seam line. To achieve satisfactory seam results, the way of finding the optimal seam path needs to be considered [23,24,25,26,27,28,29,30]. By defining a loss function and calculating the cost under different seam lines, a unique and optimal seam path can be determined. The initial seam algorithm was pixel-based [23,24,25]. To avoid seam cutting through objects, constraints of geometric structure and object detection were added, which improved the subjective quality of the results [26,27]. But pixel-based methods do not consider the semantic information in the images and their receptive fields are small. Correspondingly, superpixel partition naturally considers the boundary information of different objects, and using superpixels as the nodes can enlarge the receptive fields [28,29,30]. Yuan et al. [28] have designed the energy function of the nodes from the perspectives of color difference and texture complexity. The color difference is processed in the YUV space, while the texture complexity is represented by the Gabor filter. Peng et al. [29] stitched hyperspectral images considering both space and spectral information of HSI. Miao et al. [30] divided the overlapping area into superpixels, which are also divided into foreground and background. They introduced an energy function to prevent the seam from crossing the area where the foreground superpixels are located, thus solving the problem of lost foreground objects.

**Figure 1:**
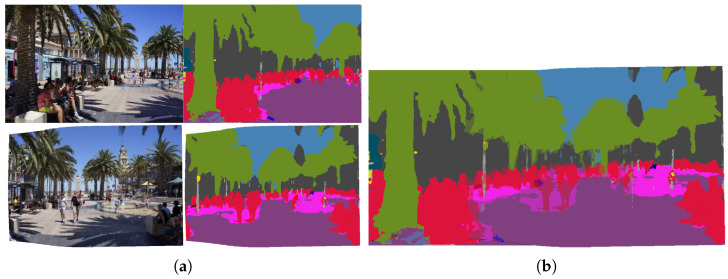
The semantic misalignment in the overlapping area after warping two images. (**a**) The two images and their semantic segmentation. (**b**) The semantic misalignment in the overlapping area.

Due to the advantages of superpixel partition, we partition each source image into superpixels to calculate the local mapping matrix to overcome the defect of grids. We also use superpixels in the seam-cutting stage to keep the texture alignment, with three costs: color difference cost, structural cost and entropy cost. However, superpixel partition cannot completely resolve the issue of semantic misalignment. In the overlapping area of the two images, due to the error of the transformation matrix, the same object’s pixel points are displaced after mapping. Figure 1 shows the detail via semantic segmentation. If the direction of the seam line does not consider semantic boundaries, it will lead to the tearing of objects in the final result, greatly affecting the subjective quality. In order to keep the objects intact, we introduce another cost referring to the result of semantic segmentation. Combining it with the previous costs results in a better quality. Experimental results demonstrate a significant improvement in subjective effects.

This paper makes three main contributions:

1. We propose an effective mapping matrix based on superpixel patches. First, we introduce a new algorithm called progressive RANSAC to collect more accurate inner points to calculate the global similarity matrix. Then, we calculate the local one on each superpixel. The mapping matrix combines global and local transformations, and hence reduces perspective distortion in both overlapping and non-overlapping areas, and improves the overall accuracy of registration;
2. A seam line algorithm based on superpixels is introduced. This algorithm assimilates various costs including color difference, structural expense, entropy cost and semantic alignment for superpixels to induce the seam line. Considering that semantic consistency is embedded within superpixels, this successfully tackles the issue of visual distortion. Through our demonstration, it is evident that the semantic alignment cost plays a pivotal role in eliminating texture ruptures;
3. The algorithm excels at accomplishing the task of multiple image stitching. We have conducted thorough experiments on numerous datasets, and the results demonstrate that in terms of multi-image stitching, the algorithm obtains excellent overall stitching results.

These contributions collectively enhance the field of image stitching by addressing issues related to perspective distortion, seam line calculation and overall visual quality in the context of multi-image stitching.

## 2. Semantic Aware Stitching Algorithm

**Figure 2:**
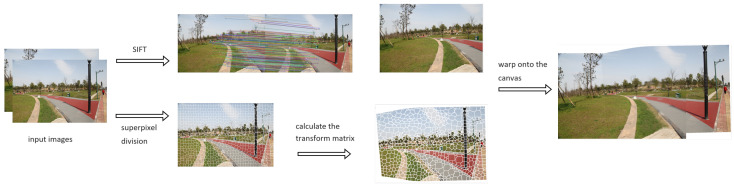
The procedure of warping image $I_{2}$ onto the $I_{1}$ plane. We calculate the warping matrices in each superpixel and warp them.

This section begins by introducing the multi-image registration process based on superpixels. Subsequently, we resegment the overlapping regions of the images into superpixels and define the cost to find an optimal seam line for artifact removal. Finally, we incorporate the Poisson blending algorithm to conceal the seam lines, rendering the images more natural. The procedure is shown in Figure 2.

### 2.1. Calculation of Pre-Registration Parameters for Superpixel Units

Our systematic stitching algorithm includes two stages. The first stage is aligning two images. In [4], Zaragoza et al. proposed a method to adjust the global by the local mapping matrices calculated on each grid. Due to the drawback of ignoring semantic information of grids, We propose a multi-image registration algorithm based on superpixel units, which estimates the local mapping matrix and global similarity transformation matrix of each superpixel block, and devises a nonlinear function to achieve the projection transformation in the overlapping region and the smooth transition of the similarity transformation in the non-overlapping region, balancing the registration while addressing the perspective distortion.

#### 2.1.1. Constructing Local Superpixel Mapping Matrix

We have two overlapping images $I_{1}$, $I_{2}$, taking $I_{1}$ as the basic plane. If the matching points are not on a single plane in the real world, or the baseline between the cameras is not negligible, a single global mapping matrix is not accurate enough. To address this, We use superpixels as the cells and the distance from each feature point to the center of the superpixel as the reference of the weight to compute the H matrix for each superpixel. We segment the image $I_{2}$ into K superpixels and first calculate the centroid coordinates of each superpixel as follows:

(1) $$C_{x}^{k}=\frac{\sum_{j=1}^{M}x_{j}^{k}}{M},C_{y}^{k}=\frac{\sum_{j=1}^{M}y_{j}^{k}}{M}$$

$\left(x_{j}^{k},y_{j}^{k}\right)$ is the coordinates of the *j*th pixel belonging to the *k*th superpixel. *M* is the total number of pixels contained in the *k*th superpixel. $\left(C_{x}^{k},C_{y}^{k}\right)$ is the center of this superpixel. The weight of the *i*th matching point is:

(2) $$w_{i}^{k}=\max\left(e^{-\frac{d_{i}^{k}}{\sigma^{2}}},\gamma \right)$$

γ is a small number to prevent the weight from equaling zero with a specific range between 0.0015 and 0.1 [4]. γ is set to 0.1 in our experiments. $d_{i}^{k}$ is the distance from the *i*th matching point to the center of the *k*th superpixel, which can be calculated as follow.

(3) $$d_{i}^{k}=\sqrt{{\left(x_{i}-C_{x}^{k}\right)}^{2}+{\left(y_{i}-C_{y}^{k}\right)}^{2}}$$

We write the weight in a diagonal matrix:

(4) $$W_{k}=\mathrm{diag}\left(\left[w_{1}^{k}w_{2}^{k}w_{3}^{k}w_{4}^{k}\cdots w_{N-1}^{k}w_{N}^{k}\right]\right)$$

Like what Zaragoza did in [4], $x^{\prime }={\left[x_{1},y_{1},1\right]}^{T}$ and $x^{\prime \prime }={\left[x_{2},y_{2},1\right]}^{T}$ are a pair of matching points. They are connected by a homography matrix H

(5) $$x^{\prime }∼Hx^{\prime \prime }$$

(6) $$\begin{array}{cc} 0_{3\times 1} & =x^{\prime }\times Hx^{\prime \prime }\\ & =\left[\begin{array}{ccc} 0_{1\times 3} & -{x^{\prime \prime }}^{T} & y_{1}{x^{\prime \prime }}^{T}\\ {x^{\prime \prime }}^{T} & 0_{1\times 3} & -x_{1}{x^{\prime \prime }}^{T}\\ -y_{1}{x^{\prime \prime }}^{T} & x_{1}{x^{\prime \prime }}^{T} & 0_{1\times 3} \end{array}\right]h \end{array}$$

h is the flattened form of the homography matrix H. In (6) only the first two rows are independent. So we stack the first two rows of the coefficient matrix of all the linear equations generated by matching points into G.

(7) $$\hat{h}={\mathrm{argmin}}_{h}{∥Gh∥}^{2},\;\;s.t.\;∥h∥=1$$

Therefore, the homography of the *k*th superpixel is:

(8) $${\hat{h}}_{k}={\mathrm{argmin}}_{h}{∥W_{k}Gh∥}^{2},\;\;s.t.\;∥h∥=1$$

**Figure 3:**
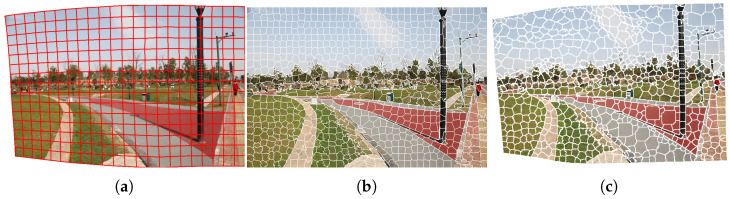
Comparison of results using grid and superpixel partition. (**a**) An example of an image after warping with grid partition. (**b**) An example of the image with superpixel partition. (**c**) An example of the image after warping with superpixel partition.

Compared with grids (Figure 3), using superpixels as units for matrix mapping can effectively preserve the edge texture in the image, making the result more natural.

#### 2.1.2. Progressive RANSAC

In the context of feature point selection using the Scale-Invariant Feature Transform (SIFT [2]) method, it is recognized that the selected feature points may be distributed across different planes. Employing the RANSAC algorithm to calculate a global similarity transformation by incorporating all feature points may introduce significant errors. Thus, a selective approach is proposed to utilize feature points from the same plane for the computation of a similarity matrix.

1. Upon obtaining initial matches of feature points, an initial RANSAC algorithm is employed with a lenient threshold (in the experiment, we chose 0.225) to eliminate only the outliers in the data;
2. For the points in the target image, a more stringent threshold (in the experiment, we chose 0.2) is applied to identify a subset of inliers, and these inliers are stored in a set, representing points on a specific plane. Since points on a plane tend to cluster together, the center of this inlier set is calculated as a representation of the plane’s center;
3. The remaining points are then filtered to remove those close to the calculated center. The filtered points are used as the new initial set for another iteration of RANSAC;
4. Steps 2 and 3 are iteratively repeated until the inlier ratio falls below 0.3. Subsequently, similarity transformation matrices are computed from the obtained sets of inliers.

The similarity transformation is a combination of an isometric transformation and uniform scaling. When there are corresponding point pairs (x,y) and $\left(x^{\prime },y^{\prime }\right)$, the similarity transformation can be evaluated by:

(9) $$\left(\begin{array}{c} x^{\prime }\\ y^{\prime } \end{array}\right)=\left(\begin{array}{cccc} x & -y & 1 & 0\\ y & x & 0 & 1 \end{array}\right)\left(\begin{array}{c} \beta_{1}\\ \beta_{2}\\ \beta_{3}\\ \beta_{4} \end{array}\right)$$

For more point pairs, LMS (Least Squares Method) can be used.

#### 2.1.3. Fusion Transformation

How to combine local homography and global similarity transformations is the main issue addressed in this subsection. It is imperative to ensure registration in overlapping regions while effectively mitigating perspective distortions in non-overlapping areas for a more natural appearance. This paper introduces a nonlinear transition approach:

(10) $${\hat{H}}_{k}^{i}=\mu_{h}^{k}H_{k}^{i}+\mu_{s}^{k}S_{i}$$

$H_{k}^{i}$ is the *k*th local homograph of the *i*th image, $S_{i}$ is the global similarity matrix of the *i*th image. ${\hat{H}}_{k}^{i}$ is the combined matrix of the *k*th superpixel in this image. $\mu_{h}^{k}$ and $\mu_{s}^{k}$ are the non-linear coefficients between zero and one. Their functions are as follows:

(11) $$\mu_{s}^{k}={\left(C_{x}^{k}-C_{0}^{i}\right)}^{a}/\left[{\left(C_{t}^{k}\right)}^{a}+{\left(C_{x}^{k}-C_{0}^{i}\right)}^{a}\right]$$

(12) $$\mu_{h}^{k}=1-\mu_{s}^{k}$$

**Figure 4:**
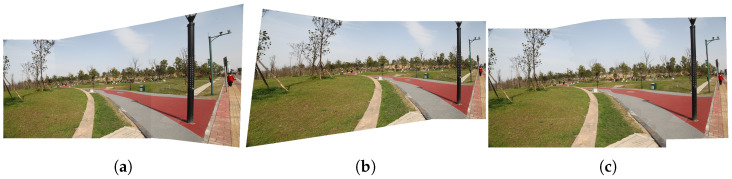
Comparison of reduction of perspective distortion. (**a**) Result of ELA. (**b**) Result of LPC. (**c**) Our result.

In (11), *a* can be a positive number between 5 and 7. $C_{x}^{k}$ is the x-coordinate of the center of the *k*th superpixel, $C_{0}^{i}$ is the minimum of the x-coordinates of the centers of all superpixels. $C_{t}^{k}$ is the width of the overlap area. As $\mu_{s}^{k}$ gradually changes from 0 to 1, ${\hat{H}}_{k}^{i}$ transitions from local homography to global similarity, effectively resolving perspective distortions in non-overlapping regions. Simultaneously, we achieve satisfactory registration in the overlapping regions, which can be seen in the comparison with two structure-keeping algorithms ELA [11] and LPC [12] (Figure 4).

For multiple images like $I_{1},I_{2},I_{3}...I_{N}$, we need to warp them onto the $I_{1}$ plane by

(13) $$\begin{array}{c} T_{n}^{k}=\prod_{i=2}^{n-1}S_{i}*{\hat{H}}_{n}^{k}=\prod_{i=2}^{n-1}S_{i}*\left(\mu_{h}^{k}H_{k}^{i}+\mu_{s}^{k}S_{n}\right) \end{array}$$

$T_{n}^{k}$ is the transform matrix of the *k*th superpixel in the *n*th image. ${\hat{H}}_{n}^{k}$ is the combined one.

### 2.2. Texture Keeping Seam Line Algorithm

**Figure 5:**
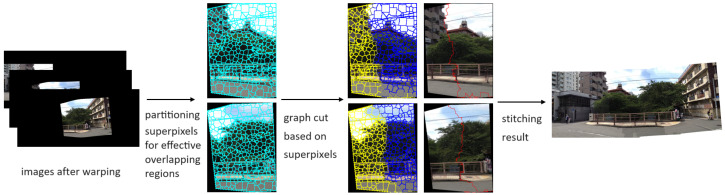
The procedure of searching the optimal seam line based on superpixels.

In this section we focus on the second stage of our algorithm, which is finding a perfect seam line to address the issue of pseudo-shadow in overlapping regions. We treat superpixel blocks as nodes in the graph and devise a similarity cost function to seek the optimal seam line. The procedure is shown in Figure 5.

In [28], the authors designed the energy function of the nodes from color difference and texture complexity. They calculated the color difference between the adjacent superpixel patches in the YUV space and the texture complexity by Gabor features. The final energy function was obtained from a self-defined norm. In our opinion, the YUV space is not sufficient to represent the accurate color distribution in terms of human eyes. So we choose the RGB and the LAB color spaces to depict the difference in color distribution. Gabor filters are suitable for representing complex textures, but they require high computational resources. If the goal is merely to describe texture differences, simpler algorithms can be employed. Therefore, we use gradient histograms and image information entropy to describe the differences in texture. The specific algorithm steps are detailed in the following text.

Let $P=I_{0}\cap I_{1}$ be the effective overlapping region after warping $I_{1}$ onto $I_{0}$. $P_{0}$ and $P_{1}$ represent the images corresponding to $I_{0}$ and $I_{1}$, respectively, in the region P. The pixel values of P are the averages of $P_{0}$ and $P_{1}$. Then, we divide P into superpixels, represented by ${\left\{S_{i}\right\}}_{i=1}^{N}$, in which *N* is the number of the superpixels in the effective overlapping region.

#### 2.2.1. Similarity Cost Function

To compare the dissimilarity between superpixels, we characterize the differential cost function through color differences, local gradients and local information entropy. Initially, a greater number of color components in the LAB color space are employed to represent chromatic aberration costs. LAB, designed based on human perception of colors, offers the advantage of perceptual uniformity, making it more consistent with the visual perception of the human eye. Constructing a 6-dimensional color layer A={R,G,B,L,a,b}, the initial chromatic aberration cost function for the overlapping regions of two images is defined as follows, $I_{0}$ and $I_{1}$ representing the corresponding superpixels in the overlapping region:

(14) $$W_{1}=\sqrt{\sum \left(I_{0}^{c}-I_{1}^{c}\right)/\left|A\right|},\;\;c\in A$$

In addition to chromatic information, we also consider structural characteristics. Due to the pronounced structural differences within regions affected by significant artifacts, conventional image gradients are inadequate. This paper introduces the concept of a gradient direction histogram for superpixels, providing a compact representation to compress information from multiple pixels. This not only condenses gradient information but also enhances noise resistance, making it less sensitive to noise and more conducive to algorithm optimization.

For each superpixel $S_{i}$, the first step involves calculating the horizontal and vertical gradients of pixels within the superpixel.

(15) $$G_{h}^{S_{i}}\left(x,y\right)=f\left(x+1,y\right)-f\left(x-1,y\right),\;\;\forall \left(x,y\right)\in S_{i}$$

(16) $$G_{v}^{S_{i}}\left(x,y\right)=f\left(x,y+1\right)-f\left(x,y-1\right),\;\;\forall \left(x,y\right)\in S_{i}$$

Subsequently, calculate the gradient magnitude and direction for a specific point within the superpixel.

(17) $$M^{S_{i}}\left(x,y\right)=\sqrt{G_{h}^{S_{i}}{\left(x,y\right)}^{2}+G_{v}^{S_{i}}{\left(x,y\right)}^{2}}\;\;\forall \left(x,y\right)\in S_{i}$$

(18) $$\theta^{S_{i}}\left(x,y\right)=\arctan\left(G_{h}^{S_{i}}\left(x,y\right)/G_{v}^{S_{i}}\left(x,y\right)\right)$$

The gradient direction is generally represented as a positive value; thus, the gradient direction can be expressed as:

(19) $$\theta^{S_{i}}\left(x,y\right)=\left\{\begin{array}{c} \theta (x,y)+\pi ,\;\;\theta (x,y)<0\\ \theta (x,y),\;\;\;\;\mathrm{others}\; \end{array}\right.$$

The gradient direction histogram has nine bins, partitioning π into nine bins. The gradient direction histogram of a superpixel can be represented as follows, with $\theta_{k}$ the angular range within the *k*th bin, $M_{p_{\theta }}$ the gradient magnitude at an angle of $p_{\theta }$.

(20) $$h_{k}^{S_{i}}\left(p_{\theta }\in \left[\theta_{k},\theta_{k+1}\right)\right)=\sum_{p_{\theta }=\theta_{k}}^{\theta_{k+1}}M_{p_{\theta }},\;\;k=0\ldots 9$$

The structural cost function based on superpixels can be expressed as:

(21) $$W_{2}^{S_{i}}=\sqrt{\sum \left(h_{k}^{S_{i}^{0}}-h_{k}^{S_{i}^{1}}\right)/\left|\theta \right|},\;\;k=0\ldots 9$$

In the equation, $W_{2}^{S_{i}}$ represents the structural cost for the *i*th superpixel, $h_{k}^{S_{i}^{0}}$ denotes the *k*th bin component of the gradient direction histogram within the *i*th superpixel in image $I_{0}$. Similarly, $h_{k}^{S_{i}^{1}}$ represents the *k*th bin component of the gradient direction histogram within the *i*th superpixel in image $I_{1}$.

In certain scenarios, the ideal seam line should align with sparse texture areas, such as smooth roads and uniform skies. These visually comfortable areas typically exhibit simpler textures. This paper introduces the concept of superpixel entropy to replace the complexity of texture variations. Viewing each superpixel as a source of information, superpixel entropy can be used to measure the randomness (balance) within each superpixel system.

The probability corresponding to the *j*th grayscale value of pixels within the superpixel information source is given by:

(22) $$p_{j}^{S_{i}}\left(R_{j}^{S_{i}}\right)=\mathrm{count}\left(R_{j}^{S_{i}}\right)/\mathrm{count}\left(R_{i}\right)$$

Here, $R_{i}$ represents the grayscale values possessed by the *i*th superpixel, and $R_{j}^{S_{i}}$ represents the *j*th grayscale value within the *i*th superpixel. The count operation denotes the statistical quantity.

The entropy of superpixel $S_{i}$ is defined as $E_{S_{i}}=-\sum_{j=1}p_{j}^{S_{i}}\log p_{j}^{S_{i}}$, where $p_{j}^{S_{i}}$ is the proportion of the *j*th grayscale value within superpixel $S_{i}$.

The cost function for defining superpixel entropy is as follows:

(23) $$W_{3}^{S_{i}}=\left|E_{S_{i}^{0}}+E_{S_{i}^{1}}\right|$$

$E_{S_{i}^{0}}$ represents the entropy of superpixel $S_{i}$ in image $I_{0}$, and similarly, $E_{S_{i}^{1}}$ represents the entropy of superpixel $S_{i}$ in image $I_{1}$.

In summary, the difference cost function for a superpixel block $w\left(S_{i}\right)$ is composed of the three aforementioned parts.

(24) $$w\left(S_{i}\right)=\frac{1}{n}\sum_{\left(x,y\right)\in S_{i}}W_{1}\left(x,y\right)+W_{2}^{S_{i}}*W_{3}^{S_{i}}$$

In this expression, the summation term represents the chromatic aberration cost function for the superpixel $S_{i}$. $W_{2}^{S_{i}}$ is the cost of entropy, and $W_{3}^{S_{i}}$ is the structural cost.

#### 2.2.2. Semantic Misalignment Cost

Despite the purpose of the previous section being to find the relatively weak texture of superpixel boundaries as the walking route for the seam line, the same semantic object is not perfectly overlaid in the overlapping area due to parallax. If the seam line happens to pass through the misaligned semantics, it will cause the stitched image to show a tear in the object, affecting subjective quality. In order to avoid this situation as much as possible, we use the semantic segmentation information of the two original images in the overlapping area given by Deeplabv3 [31] to find the part of the same semantics that causes ghosting. We calculate the proportion of the semantic ambiguity part in this superpixel as the semantic penalty term. By dynamically adjusting the gamma weight of the semantic penalty term by calculating the average of the first three penalty items, the semantic penalty term is added to the previous loss function as the weight of the nodes in the graph cut algorithm. The gamma can be adjusted based on the importance of the semantic cost.

The updated cost function can be expressed as:

(25) $$w\left(S_{i}\right)=\frac{1}{n}\sum_{\left(x,y\right)\in S_{i}}W_{1}\left(x,y\right)+W_{2}^{S_{i}}*W_{3}^{S_{i}}+\gamma *W_{4}^{S_{i}}$$

#### 2.2.3. Objective Energy Function

To ensure that the stitching line passes through the middle of the overlap area, Ref. [28] introduced another cost, typically treating it as a binary-labeled Markov Random Field (MRF) problem. The solution for the optimal seam line involves minimizing the energy function, which is composed of the data penalty term $D_{S_{i}}$ and the smoothing term $\bar{M}\left(t\right)$. The specific objective function is as follows:

(26) $$E\left(t\right)=\sum_{S_{i}\in P}D_{S_{i}}\left(t_{S_{i}}\right)+\alpha \bar{M}\left(t\right)$$

Here, α is an adjustable parameter, $\bar{M}\left(t\right)$ is calculated as follows.

(27) $$\Gamma_{U}\left(\Phi \left(S_{i}\right)\right)=\left\{\left|t_{S_{i}}-t_{S_{j}}\right|,\forall S_{j}\in U\left(S_{i}\right)\right\}$$

(28) $$\bar{M}\left(\Phi \right)=\sum_{S_{i}\in P}\langle \Gamma_{U}\left(\Phi \left(S_{i}\right)\right),\bar{w}\left(S_{i}\right)\rangle$$

(29) $$\bar{w}\left(S_{i}\right)=\left\{w\left(S_{i},S_{j}\right):=\mathrm{mean}\left(w\left(S_{i}\right)+w\left(S_{j}\right)\right)|\forall S_{j}\in U\left(S_{i}\right)\right\}$$

$t_{S_{i}}$ is the label shows from which image the superpixel $S_{i}$ comes from. $S_{j}\in U\left(S_{i}\right)$ implies that $S_{j}$ is adjacent to $S_{i}$. $\bar{w}\left(S_{i}\right)$ represents the weight of edges connecting any adjacent superpixel nodes.

Treating each superpixel in the overlap region as a node in the graph, construct the graph structure of superpixels and use the maximum flow minimum cut algorithm for solving.

When there is uneven lighting and significant exposure differences between the reference image $I_{0}$ and the target image $I_{1}$, the seam line can be quite noticeable. To achieve a more natural result after processing, we use the Poisson blending algorithm [32].

## 3. Experiment Result

**Figure 6:**
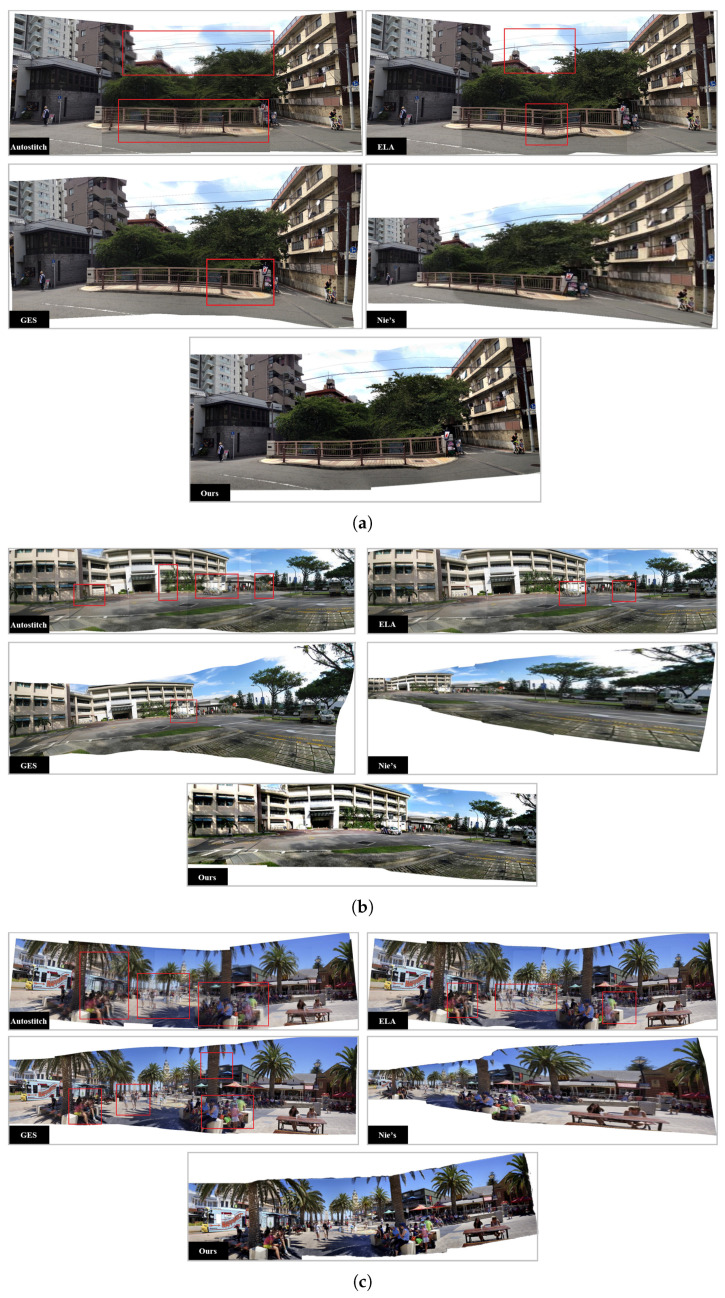

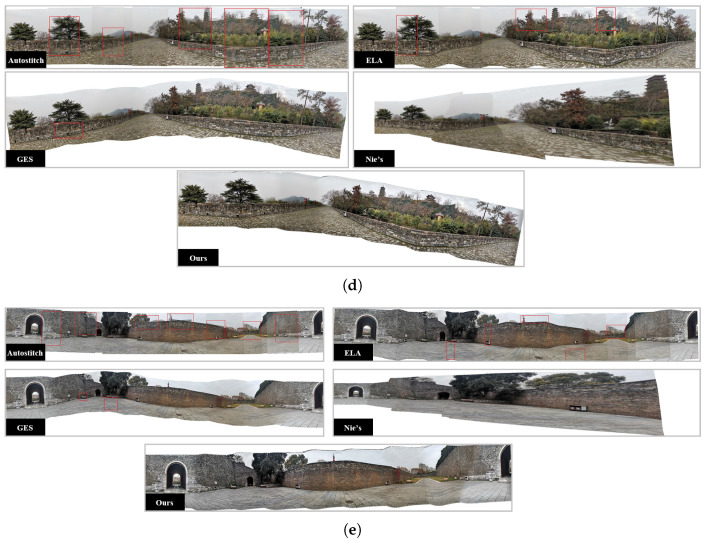
The results of AutoStitch, ELA, GES-GSP, Nie’s method and the proposed algorithm on street, carpark, school, njtemple and njgate scenes, with pseudo-shadow areas annotated using red bounding boxes; (**a**) street; (**b**) carpark; (**c**) school; (**d**) njtemple; (**e**) njgate.

**Figure 7:**
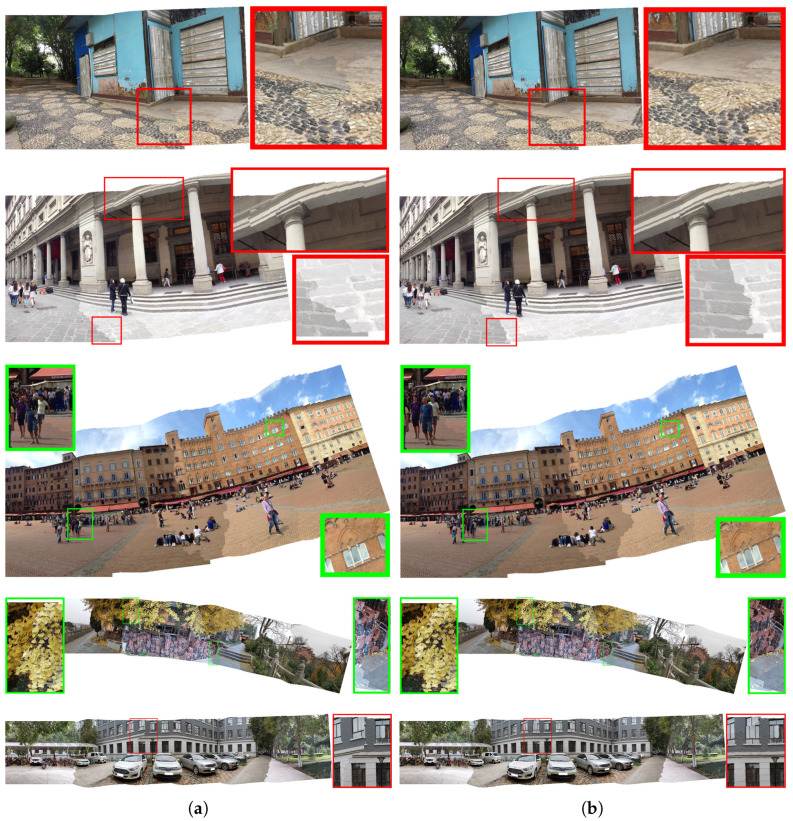
The seam-cutting results of Gabor method and our algorithm on the cabin, gallery, piazza, njyard and xdcar scenes, with semantic misalignment areas annotated using red or green boxes. (**a**) Gabor. (**b**) Ours.

The datasets we used in Figure 6 are SPHP-Street, DHW-Carpark and APAP-Train provided in the SPHP [10], DHW [3] and APAP papers [4], respectively, and our own collected datasets, Njtemple and Njgate. The SPHP-Street dataset consists of three images, the DHW-Carpark dataset consists of five images, the APAP-Train dataset consists of six images, the Njtemple dataset consists of seven images and the Njgate dataset consists of nine images. The datasets we used in Figure 7 are, respectively, the Cabin dataset from the ELA [11] paper, the Uffizi Gallery and PiazzaCampo datasets from the NISwGSP [6] paper and our own collected datasets Njyard and Xdcar. The Cabin dataset consists of two images, the Uffizi Gallery dataset consists of four images, the PiazzaCampo dataset consists of four images, the Njyard dataset consists of six images and the Xdcar dataset also consists of six images. We demonstrate the superiority of our algorithm from two perspectives: visual comparisons with other stitching algorithms and quality evaluation using average gradient and image entropy metrics.

### 3.1. Subjective Assessment

#### 3.1.1. Without Semantic Cost

We first compared our algorithm without the semantic misalignment cost (25) with other stat-of-the-art algorithms to test the effectiveness of artifact elimination. We selected 5 sets of scenes with relatively complex environments prone to pseudo-shadow occurrences for experimentation.

The results of AutoStitch [1], ELA [11], GES-GSP [13], Nie’s unsupervised method [22] and our algorithm are presented in Figure 6. The results show that our algorithm is more effective in eliminating pseudo-shadows than these algorithms. It should be noted that Nie’s method is mainly designed for stitching two images together. When the number of images to be stitched exceeds three, serious stretching deformations will occur. To make the results easier to demonstrate, we only stitched up to four images using Nie’s method.

#### 3.1.2. With Semantic Cost

Then, we add the semantic misalignment cost to the $w\left(S_{i}\right)$ in (25) to eliminate the ruptures and misalignment in textures in stitched images which cannot be addressed by the previous algorithm. We compare our seaming method with Yuan’s [28] to show our effectiveness. For distinction purposes, let’s provisionally refer to the algorithm in [28] as the Gabor method. In the first stage, we align two images using the method mentioned in Section 2.1. Then, we use our method and the Gabor method in the second stage respectively to observe the performance on the stitched textures.

Figure 7 shows the seam-cutting results of the Gabor method and our method. As a result of considering the semantic misalignment cost, our method gets better-seamed images with fewer object ruptures and misalignment, especially the ground texture in the cabin scene, the eave in the gallery scene, the person in the piazza scene, the tiles in the njyard scene and the windows in the xdcar scene.

### 3.2. Objective Evaluation Metrics

In this section, a comparison is made based on the objective quality of the results, utilizing two metrics: average gradient and entropy. The average gradient reflects the expressive ability of image detail contrast, indirectly indicating clarity, while entropy reflects the average information content in the image.

First, we calculate the horizontal gradient $G_{h}\left(x,y\right)$ and the vertical gradient $G_{v}\left(x,y\right)$.

(30) $$G_{h}\left(x,y\right)=f\left(x,y\right)-f\left(x-1,y\right)$$

(31) $$G_{v}\left(x,y\right)=f\left(x,y\right)-f\left(x,y-1\right)$$

f(x,y) represents the grayscale of (x,y).

Then, the average gradient *G* of the image whose size is M×N.

(32) $$G=\frac{1}{M\times N}\sum_{i=1}^{M}\sum_{j=1}^{N}\sqrt{\frac{{\left(G_{h}\left(x,y\right)\right)}^{2}+{\left(G_{v}\left(x,y\right)\right)}^{2}}{2}}$$

The information entropy of the image *H* is represented as (33), where $p_{i}$ represents the proportion of pixels in the image with a grayscale value of *i*.

(33) $$H=\sum_{i=0}^{255}p_{i}\log p_{i}$$

Table 1 presents the statistical results of the four algorithms using average gradient and entropy for the five sets of images: street, car park, school, njtemple and njgate.

Average gradients are mostly distributed between 9 and 15. In these five scenes, our algorithm exhibits improvement compared to the others, with notable enhancements observed in the street scene, and minor improvements in the car park, school, njtemple and njgate scenes. The information entropy is mostly distributed between 7 and 8. Our algorithm shows improvement over the other four algorithms in all five scenarios.

Table 2 presents the statistical results of algorithms with Gabor seaming and our seaming method using average gradient and entropy for the five sets of images: cabin, gallery, piazza, njyard and xdcar.

We can see from Table 2 that our method is slightly better than Gabor method in terms of average gradients and information entropy.

## 4. Conclusions

In order to fully utilize the information in the images and make more precise local adjustments to the homography matrix, we chose superpixels as the local mapping units. We initially compute the local mapping matrix for each superpixel based on the inliers obtained from RANSAC. Subsequently, we compute the global similarity transformation for each input image and construct adaptive nonlinear transformation functions for each image. This ensures a smooth transition between the local homography of superpixels and the global similarity transformation. Finally, a reference image is chosen, and each image is mapped onto the canvas, resulting in the pre-registered outcome.

To address the various artifacts introduced by parallax, a superpixel-based artifact removal algorithm is proposed. It starts by performing superpixel segmentation on the effective overlapping region. To measure the color difference of superpixels, a six-layer color model is constructed to calculate the color cost function. To measure the structural difference of superpixels, the gradient orientation histogram for each superpixel is computed to construct the structural cost function. Subsequently, the cost function of superpixel entropy is introduced to adjust the coefficients between color and structure. The cost functions mentioned above can solve the artifact issue well but they fail in semantic misalignment. Therefore, we add the semantic cost to adjust the path of the seam line. Finally, an objective energy function is constructed, treating each superpixel block as a node in the graph model. The graph model is solved using the max-flow algorithm to obtain the seam lines. Poisson blending is then introduced to eliminate the seam lines. A series of experiments are conducted, directly comparing the results with AutoStitch, ELA, GES-GSP and Nie’s algorithms. The quality of the results is evaluated using the metrics of average gradient and information entropy, confirming the effectiveness of our algorithm in addressing artifact problems.

## Figures and Tables

**Table 1:** The objective evaluation results for AutoStitch, ELA, GES-GSP and our algorithm.

	Average Gradient				Information Entropy			
	**AutoStitch**	**ELA**	**GES-GSP**	**Ours**	**AutoStitch**	**ELA**	**GES-GSP**	**Ours**
Street	6.8251	6.9325	8.1026	8.7826	7.2013	7.3083	6.8795	7.3400
Car Park	9.9927	10.8231	12.8243	13.0221	7.6845	7.7067	7.8383	7.9824
School	12.8547	13.0881	13.8988	13.9745	6.8441	7.1757	7.1706	7.3203
Njtemple	9.2167	9.7643	9.5043	9.8415	7.5250	7.6383	7.243	7.6538
Njgate	13.3862	13.8775	14.4181	14.7217	7.1641	7.3967	6.5605	7.4439

**Table 2:** The objective evaluation results for Gabor and our algorithm.

	Average Gradient		Information Entropy	
	**Gabor**	**Ours**	**Gabor**	**Ours**
Cabin	4.5918	4.6055	6.0899	6.0919
Gallery	4.9803	4.9853	5.2125	5.2146
Piazza	4.5749	4.5843	3.5997	3.6003
Njyard	8.0866	8.1108	5.756	5.7565
Xdcar	10.4082	10.5819	5.9043	5.9053

## Data Availability

Data are contained within the article.
